# Age, Period, and Cohort Effects on Literacy Skills across Life Stages

**DOI:** 10.1093/geroni/igab046.3070

**Published:** 2021-12-17

**Authors:** Takashi Yamashita, Thomas Smith, Phyllis Cummins

**Affiliations:** 1 University of Maryland, Baltimore County, Baltimore, Maryland, United States; 2 Northern Illinois University, DeKalb, Illinois, United States; 3 Miami University, Oxford, Ohio, United States

## Abstract

Literacy skills are essential adult competencies for economic, social, political and cultural participation, which are linked to higher quality of life. Literacy skills are known to be lower for older age groups. However, relatively little is known about cohort and period effects, which provide clues to the sociohistorical impacts on literacy, in addition to the well-known age effects, over the life course. This study analyzed three nationally representative cross-sectional survey data of the U.S. adults at five time points, from the 1994 International Adult Literacy Survey (IALS), 2003 Adult Literacy and Life Skills Survey (ALL), and the 2012/2014/2017 Program for International Assessment of Adult Competencies (PIAAC). The total analytic sample was 17,450 adults age between 18 and 65 years old. The literacy measures were re-scaled (0-500 points) to be comparable across the surveys. An age-period-cohort hierarchical linear model (i.e., cross-classified random effects model) was constructed using the Bayes estimator. Individuals were cross-classified based on 14 five-year birth cohort and 5 periods (survey years) information. Results showed that literacy skills improved [95% credibility-interval (CI) for linear effect of age = (0.31, 1.07), but the rate of improvement declined over time, faster rates of decline in later life stages [95% CI for quadratic effect of age = (-0.17, -0.09)]. Additionally, the notable variability across the cohorts and periods [95% CI variances = (5.34, 52.52) and (2.30, 172.01), respectively] were identified. Possible explanations for the identified age, period and cohort effects on literacy and implications for adult competencies in later life are evaluated.

